# Social Impact of a Transformative Service-Learning Experience in a Post-conflict Setting

**DOI:** 10.3389/fpsyg.2020.00047

**Published:** 2020-01-23

**Authors:** Lina Trigos-Carrillo, Laura Fonseca, Natalia Reinoso

**Affiliations:** ^1^Department of Psychology of Development and Education, School of Psychology, Universidad de La Sabana, Chía, Colombia; ^2^Department of Social and Organizational Psychology, School of Psychology, Universidad de La Sabana, Chía, Colombia

**Keywords:** transformative service-learning, post-conflict setting, cultural humility, undergraduate education, psychology education, psychology

## Abstract

In the context of the 2016 Peace Agreement signed between the Colombian government and the FARC-EP (Fuerzas Armadas Revolucionarias de Colombia-Ejército del Pueblo), several challenges for society and academia have emerged: (1) overcoming the gap between the rural and urban settings, which has been one of the roots of the Colombian armed conflict, and (2) training psychologists and transforming traditional educational practices, which have not been designed to fulfill community needs in a post-conflict setting. One of the strategies from academia to overcome these difficulties is to create alliances with rural communities where students learn key competences to foster a horizontal approach while actively working with the community. In the region of Caquetá, Colombia, two Territorial Spaces for Training and Reincorporation (ETCR) were created in order to provide a space for former guerrilla members’ reintegration to civil society. In the ETCR Héctor Ramírez, 27 students and two faculty participated in a service-learning project (2 weeks in December 2018 and two in June 2019) where they engaged in local daily practices and social projects based on the community’s prioritized needs. The aim of this study was to analyze the learning process of undergraduate psychology students in this community psychology service-learning project in the context of peacebuilding in Colombia. This study is grounded in a Participatory Action Research (PAR) approach and data collected include reflective narratives and video diaries by students before and during the course, and two focus groups after the experience. Findings suggest that students who participated in the experience are in the process of developing cultural humility, through affective understandings and the consolidation of communities of practice that include the former guerrilla members and their knowledges. Preparing psychologists to lead peacebuilding and reconciliation processes is of importance to the field because the professional competencies gained in this context surpass the professional practice as they become part of the students’ abilities as citizens. The social impact is twofold: the students learn to create partnerships where purposes are co-constructed and trust-based, while the community takes the lead of their processes creating alliances with an academia that recognizes their knowledge and practices.

## Introduction

Colombia is currently amid the implementation of a peace agreement with one of the oldest guerrillas in the continent, the FARC-EP. Three years after the signature of the peace agreement, Colombia is still struggling with its implementation, mainly because of the lack of resources and political will of certain members of the government. This has resulted in a recent upraising of a group of dissidents of the FARC who have returned to the arms. In this scenario of potential conflict with the ongoing reincorporation of almost 10,000 former guerrilla members (ARN, 2019), there is an urgent need for the consolidation of citizen trust in the process, as well as building alliances between former guerrilla members who have decided to respect the peace agreement and society. In this context, there is an emergence of societal challenges for peacebuilding and reconciliation. Challenges are not only global, in terms of laws and policies; at the personal level, people also require a critical reflection of personal positions regarding the internal armed conflict of the country toward reconciliation (Oettler and Rettberg, 2019).

Historically, there has been a division between urban and rural areas in Colombia (Rettberg, 2019) with different effects: (1) the access to university is more likely for urban students than for rural ones, which results in a lack of professionals in the rural settings (Departamento Nacional de Planeación, 2015); (2) the urban areas, mainly the capital cities, have not been directly affected by the armed conflict and, therefore, the majority of urban youth is not as involved in the impact of the armed conflict as youth in the countryside (Oettler and Rettberg, 2019); and (3) the professionals that reach rural areas are not prepared to deal with the structural barriers linked to the socio-historic factors underpinning a post-conflict scenario (Vega and Bajaj, 2016).

Populations that have experienced the armed conflict and violence in Colombia suffered negative consequences on their mental health (Bonilla-Escobar et al., 2018). Research on mental health interventions in post-conflict contexts posits the need of trained psychologists who can support dissemination and implementation of evidence-based effective health care approaches (Murray et al., 2014). Further, some communities require community interventions that consider their cultural characteristics and their coping mechanisms (Bonilla-Escobar et al., 2017). In particular, post-conflict contexts require professionals who are prepared to work with culturally diverse populations, recognizing the victim’s multiple local knowledges and cultural practices, and offering an empathetic and rigorous service (Osorio-Cuellar et al., 2017). These mental health needs pose challenges for psychology education and psychology training programs in higher education.

As a result, there have been institutional (Diazgranados et al., 2014; CNMH, 2015) and scholarly (Gómez-Suárez, 2017; Sánchez-Meertens, 2017; Díaz-Gómez et al., 2019; Oettler and Rettberg, 2019) efforts to promote peacebuilding processes by including them as part of the curriculum. In the case of universities, the approach has focused on formal settings, where the students have affective and emotional development by exploring the Colombian History (Gómez-Suárez, 2017; Corredor et al., 2018; Díaz-Gómez et al., 2019; Oettler and Rettberg, 2019). The importance of informal settings has been also highlighted, particularly by including visits to museums and media analysis as part of the curriculum (Corredor et al., 2018); however, this approach is yet to be further explored in the literature. In general, there is still a lack of analysis on the impact of peace pedagogies in the country (Sánchez-Meertens, 2017).

Bearing in mind that the educational proposals in terms of peacebuilding in the universities’ curriculum is currently focused on formal settings, it is important to address the need to implement alternative approaches that provide students with real-life scenarios where they can incorporate theory and practice. Research on students’ perceptions about the ex-combatants has shown that young adults in private universities are more likely to forgive former guerrilla members (López-López et al., 2016). As a result, in this sociopolitical scenario, a service-learning approach (García-Romero and Lalueza, 2019) can be useful to further promote safe spaces for students to engage in dialogue with historically distant groups. This approach can provide the basis for developing courses that incorporate not only the theory, but specific scenarios where students can critically think about their own positioning, practices and beliefs, and the impact that communities can have on them. This is also in line with current Colombian regulations (Decree 1030, 2019) which deem it crucial to engage psychology students in real-life scenarios where they learn to provide creative and transformative solutions to local, regional, and local problems (Corte Constitucional de Colombia, 2019). In addition, Law 1090 of 2006, which regulates psychology practice and education in Colombia, requires psychology programs to form practitioners who can work toward the development of communities under culturally responsive practices and ethical behavior (Corte Constitucional de Colombia, 2006).

Transformative Service-Learning (TSL) is a teaching strategy in which students are involved in a community that is different from their own (Jones and Abes, 2004) and it involves a process of individual and social transformation derived from this experience (Naudé, 2015). Moreover, it is a curricular activity (bearing credits) that requires students and faculty to work with the community (Bringle et al., 2016) with regular and structured spaces for reflection and meaning attribution to unknown cultures (Naudé, 2015; Bringle et al., 2016). The impact of service-learning in higher education has presented positive effects on student’s formation (Chan et al., 2019). In the case of psychology, the implementation of service-learning classes has proven to be effective in developing key competences in students (Li et al., 2016), particularly the ethical and social responsibility in a diverse world, which is one of the goals of the APA Guidelines (Bringle et al., 2016) and is required by the Law 1090 of 2006. Psychology education has incorporated TSL; for instance, teaching in schools in South Africa (Naudé, 2015). However, there is only one reported study analyzing the role and benefits of service-learning teaching in a post-conflict scenario, for both citizenship and intercultural education in Serbia (Dull, 2009). Consequently, there is a need for research on training future psychology professionals who can recognize their role as peacebuilding facilitators in post-conflict contexts. The aim of this paper is to analyze the learning process of undergraduate psychology students in a TSL project. This TSL experience took place in one of the 24 Territorial Spaces of Training and Reincorporation (ETCR in Spanish) in Caquetá, Colombia, where former guerrilla members and their families are currently building a community in peace.

### Transformative Service-Learning

In order to foster critical reflection over the challenges of a post-conflict context, TSL can be useful to create enabling spaces for training future psychologists to become critically aware of the challenges people face after the end of an armed conflict. The axis of the TSL approach is the experience of alterity by the students, which implies an identity transformation based on crossing sociocultural borders (Naudé, 2015), as well as a critical reflection on these alternative worldviews (Gómez-Suárez, 2017). The emphasis on transformation highlights the fact that students experience a paradigmatic shift (Taylor, 2007), understood as a process of transforming the structures of assumptions through which we give sense to experience. A paradigmatic shift has three key elements involved: the first element is a *disorienting dilemma*, or “an activating event that typically exposes a discrepancy between what a person has always assumed to be true and what has just been experienced, heard or read” (Cranton, 2002, p. 66). Disorienting dilemmas can lead to transformation when they come with critical reflection or self-reflection, which is the second element of the process. Cranton (2002) defines critical reflection as, “the means by which we work through beliefs and assumptions, assessing their validity in the light of new experiences or knowledge, considering their sources, and examining underlying premises” (p. 65). The third element is the emotional process, emphasized by García-Romero and Lalueza (2019) as a crucial contribution from Kiely (2005) to the model; this key element is involved in the *contextual cross-bordering*, the experience of *dissonance* and the particular form of knowledge implied in the *personalization of the other* occurred as a result of relationships and bonding. According to Naudé (2015), as students build relationships with the community, peers, and faculty, they gain a comprehension of the emotions involved in the experience, an *affective understanding*, which is a critical component in the transformative process. In line with Mezirow (2000), she states, “high-intensity dissonance leads to intense emotions, the questioning of the self and society, and long-term adaptations. Thus, high-intensity dissonance facilitates true transformative learning” (Naudé, 2015, p. 86).

In sum, TSL provides the pedagogical tools to engage students in social transformation in unfamiliar contexts, such as post-conflict settings, and has been used in training cultural competences in health and education professionals. In turn, the concept of cultural humility informs the analysis of this TSL experience, considering the preliminary findings related to experiences of introspection, cultural awareness, and the learning of a specific role as psychologists.

### Cultural Humility

Recently, UNESCO (2017) made a call to reinforce the development of intercultural competences in all scenarios of formal, non-formal, and informal education with the goal of *learning to live together and in peace* in a globalized world. However, no consensus has been reached about the *know-how, know-what, and know-who* required to live together, maximizing the advantages of cultural interactions (UNESCO, 2017).

From the beginning, cultural competences were aimed to overcome health inequities related to race and ethnicity by forming knowledgeable professionals about the others and their culture. However, cultural competence places the power in the professional’s hands, and as a result they are the ones who define the problem and decide how to solve it (Isaacson, 2014; Hook et al., 2017). Moreover, expertise on the others and their culture does not necessarily result in culturally safe interactions (Brascoupé and Waters, 2009). In fact, expectations based on expertise may interfere in the intercultural learning process because the fear of being perceived as incompetent surpasses the possibilities of learning by recognizing what we do not know about the others (Hook et al., 2017).

Cultural humility is a value that informs one’s culture and worldviews (Abbot et al., 2019), which is crucial in a multicultural framework (Hook and Davis, 2019). Instead of an achievable competence, it is a lifelong process with intrapersonal and interpersonal dimensions (Hook and Davis, 2019). The first involves cultural consciousness, cultural limitations, and self-reflection on one’s privilege and power (Yeager and Bauer-Wu, 2013; Fischer-Borne et al., 2014), and awareness about the impossibility to fully understand other worldviews (Hook and Davis, 2019). The latter involves recognition of the others’ knowledge and expertise (Isaacson, 2014), and respect and curiosity toward their culture and history (Hook and Davis, 2019), which leads to supportive interaction, honest working relationships, and caring (Foronda et al., 2015).

In psychology education, cultural humility has not played such an important role as in other health professions, but lessons learned in psychotherapy training can be used to teach how to work with communities in educational and research contexts (Abbot et al., 2019). Different teaching strategies have in common the importance of modeling this value by self-reflection on one’s culture, limitations and privilege; showing openness and curiosity to others, honoring their identity, and facilitating educational spaces in which humility can occur (Abbot et al., 2019).

The concept of cultural humility served as a framework for this study as we aimed to understand how students and teachers worked toward a transformation on their conceptions about and understandings of former guerrilla members, their families, and their recently formed communities.

## Methods

### Study Context

This study was conducted in a psychology program in a private university in Bogota, Colombia. As part of the undergraduate curriculum, a TSL course called “Community Psychology Applied to Post-conflict Settings” was designed in alliance with the ETCR Héctor Ramírez (ETCR-HR) in Caquetá, Colombia, where former guerrilla members and their families are currently living and constructing a community. The design and approval of the course was a challenging task given the university’s mistrust and unawareness of communities created by former guerrilla members. This could be due to the historical disconnection between urban and rural settings, particularly in areas directly affected by the armed conflict.

The main objective of the course was to apply the values and foundations of transformative community psychology to nurturing, bridging, and bonding social capital through collaborative interactions with community stakeholders. The course was 14 days long, with 3 days of teaching on campus and 11 days in the ETCR-HR in Caquetá. Students lived in the former guerrilla members’ households during the visit and shared their daily activities as part of the TSL course.

Caquetá is located in the South of Colombia, in the Amazon piedmont. This region is characterized by being a historical stronghold of the FARC-EP guerrilla, particularly due to the vast mountains and rainforest where they used to live. As such, this is mainly a rural area which has been neglected by the State. From the main airport of the region, the students and teachers had to travel 1 h by bus to the village. The ETCR-HR is a community formed by 90 former guerilla members and their families (around 250 people). This territory was initially rented to the community by the State and it is currently owned by its inhabitants as a result of the implementation of agricultural self-income generating projects. In front of the Amazon piedmont, lines of colorful houses, crops, and roads have become the niche of a peaceful community that struggles to build peace in a polarized country.

### Research Design

This study was designed following a Participatory Action Research (PAR) methodology, in order to plan, monitor, and evaluate the students’ learning process and interactions before, during, and after the implementation of the TSL course. PAR methodology followed a process of (1) a collective recognition of all the participants involved (students, teachers, and community stakeholders), their concerns, needs, and current activities through the consolidation of a public sphere; (2) the participatory planning of actions to collaborate with the community processes; (3) the implementation, monitoring, and evaluation of the project; and (4) a collective reflection on the whole process (Kemmis et al., 2014; Loewenson et al., 2014).

The PAR cyclical and spiral process was developed for the TSL courses held in December 2018 and in June 2019. The analysis we present here also involves the use of a PAR to evaluate the impact of the TSL course in the training of psychology students in both versions of the course. Three moments of the process were analyzed for each version: on-campus training (before traveling to ETCR-HR), fieldwork in ETCR-HR, and the reflections of the team 1 month after they finished the TSL course. This analysis acknowledges the spiral and cyclical nature of a PAR approach, as we consider transformation as a continuous and incremental process.

### Study Population, Sampling, and Recruitment

The selection process for the two versions of the course included an open call to all the undergraduate students of the Psychology Program. This call was sent *via* email and social networks, as well as printed flyers on campus. The students who participated in December were selected based on their academic achievement and their previous participation on the undergraduate student’s research seedbed group called “Social Action and Communities” (SRG). For the June version, considering suggestions from the December group, students participated in an assessment center with tasks to evaluate teamwork, creativity, and assertiveness, as basic skills required for the course. After they were selected, students and parents signed an informed consent for their participation in the TSL course and in this study.

### Participants

Selected participants included 16 students in the December version (Group A) and 11 in the June version (Group B). In both TSL courses, two faculty members with experience in social and community psychology oversaw the course and fieldtrips. Group A was composed mainly of senior psychology students (12 women and 4 men) who had worked closely in the SRG; the mean age was 20 years old. Group B was formed by second year students (seven women and four men); only three students were part of the SRG and the mean age was 19 years. Researchers conducted an oral informed consent process with the students explaining the nature of the project, the extent of their participation, and the possibility to withdraw at any time. Students had time to ask questions regarding the project and then they agreed to participate orally, and it was recorded in a recording device. We changed all names by pseudonyms to protect the students’ confidentiality.

### Data Collection

Data collection varied from one version to another given the evolution of the course through time (Table 1).

**Table 1:** Data collected for each group.

Group	Type of data collected		
	Pre-departure	During the SL course	After arrival
Group A December 2018	Narratives about expectations	Individual narratives of the experience	Focus group
		Group reports of daily activities	
Group B June 2019	Individual and collective timeline	Audiovisual reports	Focus group
		Video diaries	

#### Data Collected on Campus (Before Traveling)

Students were part of different training classes and reflection activities to be prepared for fieldwork.

##### Narratives About Expectations

In group A, each student wrote their expectations before traveling to the ETCR-HR in a short paragraph.

##### Timeline

In group B, students had to collectively construct a timeline of Colombia’s history and, then, each one had to locate their own history within the timeline. The emphasis of this session was on the reflections about the relationship between the personal stories and the country’s history.

#### Data Collected on Site

During the 11 days of fieldwork in the ETCR-HR, students had to write down different types of texts as a way to reflect about their own experience.

##### Narratives About Significant Experiences

In group A, a book was written based on the students’ and community members’ experiences; chapters were written individually by the students and community leaders. The design of the book was co-constructed with the participants, as they chose the subject they wanted to explore, based on the most significant situation or event they experienced there. Sixteen narratives were selected for the purpose of this study. Additionally, participants were assigned into groups of three and each night one group had to write a small report of the events of the day.

##### Audiovisual Data

In group B, students recorded video diaries of their experiences at the end of the day. As in the December visit, they were assigned in groups of three to report the events of the day. Additionally, as part of the final product of this visit, participants had to construct a photo report of a subject that caught their attention during the course. The presentation of the photo reportage to the community on the final day was recorded by one of the professors.

#### Data Collected on Campus (After Traveling)

After 1 month of arrival to Bogota, students were invited to analyze the process.

##### Focus Group

In both versions of the course, students participated in a focus group to analyze the TSL experience following the spiral process of PAR. The dialogical spiral was drawn in a cardboard with the four moments of the cycle: collective recognition, participatory planning, action, and reflection (Kemmis et al., 2014; Loewenson et al., 2014). Students identified the activities that were crucial for each moment, wrote them on post-its and pasted them on the cardboard. A discussion was developed based on the collective construction of the cycle. Five students participated in the group A and 9 in the group B focus groups.

### Analytic Approach (Data Analysis)

Data collected were analyzed following the procedures of initial, focused, and axial coding proposed by Charmaz for critical qualitative inquiry (Charmaz, 2014, 2017). The process involved a team-based, inductive, bottom-up coding in order to develop a rigorous analytic process (Cascio et al., 2019). The authors of the paper and two research assistants had weekly meetings to jointly analyze the transcripts and videos of the different groups. Additionally, the coding process was informed by the TSL and cultural humility theories (Taylor, 2007; Abbot et al., 2019).

Initial coding included line by line coding (Charmaz, 2014). Emerging themes were related to previous worldviews about the former guerrilla members and their communities, paradigmatic shifts during fieldwork, and transformations after the TSL course. We found some *in vivo* codes, such as “getting out of the bubble” and “looking at the reality.” Focused coding (Charmaz, 2014) was more significant than the initial codes. In this stage, we concentrated on analyzing transformation through time and the emotional understandings gained by students during and after the experience. We also looked at salient codes that emerged during the initial coding in Excel tables. Finally, we conducted axial coding (Charmaz, 2014), where we organized data into major categories and subcategories. Final categories included: *(1) The other one as different, (2) developing affective understandings, (3) developing cultural humility for community partnerships, (4) forming communities of practice,* and *(5) from peace agents to self-reflective practitioners.* These categories are developed in the next section.

### Trustworthiness

To grapple with preconceptions about the students and the community of former guerrilla members, we developed methodological self-consciousness (Charmaz 2017), or a deeply reflective gaze on ourselves and the research process. This was achieved as a result of the characteristics and experiences of the researchers. One of the authors was not a professor of the course but had conducted research fieldwork in this community, another author was one of the teachers of both courses (groups A and B), and a final author was the teacher of group A. The three authors have had experiences of alterity and significant intercultural interactions; one of them working with indigenous communities in Colombia, and the other two living abroad for extended periods of time.

In order to guarantee trustworthiness, the three researchers created field journals, triangulated data sources and data analyses, conducted frequent peer debriefing, maintained audit trail, and mounted other safeguards (Lincoln and Guba, 1985). Because two members of the research team were also teachers of the two TSL course groups, all data analyses were carried out after the end of the two versions of the course, data were analyzed independently and in group, and the third researcher frequently brought an outsider perspective to the analysis.

## Results

The aim of this study was to analyze the learning process of undergraduate psychology students in a community TSL course in the ETCR Héctor Ramírez in Caquetá, Colombia, where former FARC guerrilla members and their families are constructing a community in the context of peacebuilding. Results from this study suggest that undergraduate psychology students experienced a positive social impact during and after the TSL course, as they developed at different levels and in different degrees intercultural competences. In this study, we focused on cultural humility as a framework to transform one’s worldviews and to understand others and self, while building a different kind of relationship with those formerly constructed as other. We argue that a TSL experience as the one described in this study helped undergraduate psychology students revalue their own preconceptions about people who were involved in the Colombian armed conflict and their role as practitioners and citizens in the context of peacebuilding. Further, affective understandings allowed the personalization of the other and personal transformation. It emerged through bonding and reflection processes between students and local actors and their families, with whom they shared everyday life. The process of developing cultural humility through the pedagogical strategy designed in the TSL project shapes the specific role of a psychologist able to build bidirectional partnerships with community members in contexts of peacebuilding.

### The Other One as Different (Conceptualizing the Idea of the Other)

Initial reports by students started with a notion of “understanding the other,” which for them entailed sharing time with people they did not know and assumed as different. Narratives about their expectations presented an idea of the former guerrilla members as people to be known and discovered by them as part of the experience:

I chose the course because of the different type of experiences it offers, the new opportunities to know yourself and to share with others. My main expectations are: To discover a way of being, living and interacting with the ETCR community and to contribute in any way I can. (Marcela, 22 years old 7th semester, group A, Narratives about expectations)

In the case of the group B, the individual and collective timeline allowed them to identify the plurality of life stories that classmates had regarding the armed conflict:

The first day we met in class [before traveling to Caquetá], when we were telling our individual stories, it was very shocking to know that some were related to the military, and then I understood that each of us has different realities and that the experiences that we were going to have were going to be completely different. (Diana, 19 years old, 7th Semester, group B, timeline)

Nevertheless, once students started living with the community, narratives of the experience started to highlight the importance of sharing stories in daily activities as a mutual process. Then, students began to understand that community was not only an object to be analyzed but a subject who was also interested in the stories they brought from the capital city, as private-university students. Students reported the initial difficulty to engage in conversations with the community members, particularly because of the silence over the questions they were asking. However, at the end of the process, in the focus group discussion, they recognized their own role as “the other” for the community, which entailed acknowledging that not only they as students had preconceptions about the former guerrilla members, they were also being considered as different and, therefore, a dialogical relationship had to be built:

As I got closer to the designated date place, I still had many thoughts. I felt like a foreigner, but that feeling dissipated quickly [...] Our chat was absolutely natural, and it was quite peculiar. It amazed me how, in this case, I had turned into the interrogated. This was one of the few moments in my life where I was the one sharing the anecdotes and urban sensations of a student. (Juan, 20 years old, 8th semester, group A)

Undergraduate psychology students had preconceptions of the former guerrilla members based on what they saw in the media – because they had never interacted with one before. However, as they shared time with the community, these preconceptions changed. Further, they realized they were represented as “the other” by the community. The process of getting to know one another was mutual: students from the capital city and former guerrilla members.

### Developing Affective Understandings

The process of peacebuilding in Colombia has been emotional for most Colombians (signing the peace agreement after more than 40 years of armed conflict and after years of expectation). As predicted, the encounter with former guerrilla members also brought an intensity of emotions to the students because they had not experienced something like this before. During the TSL course, students reported experiencing emotional struggles as part of the process of the encounter. For most students of group A, the affective understandings came more easily, along with the critical group reflections around the other as different, which resulted in the personalization of the other, the acceptance of difference, and the emergence of a shared identity:

I understood that we’re not the same. We will never be, and that’s ok. Actually, being different is what allows us to grow, build and, in some way, re-define the circle of “us,” including the wrongly so-called “others.” In spite of not being the same, the abyss I imagined when traveling in the “chiva” (rural bus) is more like a crack than the huge abyss I once saw. The once clear abyss disappeared in front of my eyes and of the gaze of that distant observer, who now reflected compassion and acceptance. (Pablo, 20 years old, 8th semester, group A, Individual narrative of the experience)

Some students of this group experienced emotional struggles during their time in Caquetá. One student reflected specifically over the continuous fear she experienced, which started before arriving to the territory with the remembrance of the former presidential candidate Ingrid Betancourt taken as political prisoner in the region of Caquetá many years ago:

Each of the eleven days I shared in the ETCR was a game of emotions. I have the opportunity to narrate an encounter, not a war. I am lucky I had the shake of a hand, not a bullet; to share a home and not a cage. Fear was the reason I came and maybe a bit of it goes back with me. (…) Monsters stop being so as soon as they walk out of the darkness, and you can see their faces. When the encounter allows comprehension and hand shaking in the difference. I am not coming back with answers or truths; I am not leaving believing I know them or that the 53-year-old reality is solved. I leave being certain that reconciliation exists here, and it needs constant work. (Sandra, 9th semester, group A, Individual narrative of the experience)

In the case of group B, emotional struggle was related to the mixed messages students faced before traveling to Caquetá: (1) families were concerned over their safety and the possibility they would become left-wing advocates, (2) prejudice over the former guerrilla members, and (3) the students’ own desire to go despite everything they had heard. Once they were in the field, students reported a dilemma about the positive experiences they had with the community, while at the same time having a hard time understanding the different values they hold. As a student stated, “We thought they could be humbler about their process and their current projects, as they are acting as if nothing happened. How can you justify people being killed in the past?” (Jessica, 19 years old, 6th semester, group B, Focus Group). For those students who did not report such mixed messages, the confusion appeared when they saw their fellow classmates struggling:

I was very confused because I thought: Am I doing this the wrong way? No one else said they were not struggling so I was questioning myself: Am I doing this the wrong way? Should I not put their history in brackets to understand other possible views? Then I realize that we are all different and we live the experience differently. (Marcela, 19 years old, 7th semester, group B, focus group)

In both groups, students reported one key aspect to overcome emotional struggling and to each affective understandings: the daily gatherings organized as part of the course, which took place in the mornings to plan the activities of the day and at night to reflect over their process and what happened during their interactions. For the students, having specific reflecting spaces as part of the course was essential to share their emotions, concerns, questions, and progress throughout the course:

I think that the first night was very important for us and every night that we gathered was very meaningful for us. I think it was because it was the moment you had to organize all the information you had and we all talked and talked and talked, we didn’t stop, we talked for hours every night, and it was very important, right? (Sonia, 19 years old, 7th semester, group B, focus group)

Additionally, students argued that daily interaction in their host houses also allowed them to work through the dilemmas, confusions, or struggles during the course:

I think there is planning at the micro level: we planned to wake up early to help with the chores, or to talk to the family, or to teach English to our landlord; it was a very important system in order to live with the others. (Catalina, 19 years old, 5th semester, group B, focus group)

Students experienced complex emotions before and during the encounter with former guerrilla members. They had different expectations based on their experiences and histories. However, affective understanding was important to achieve comprehension of one’s feelings and of others. Affective understanding through relationship establishment configured the role required for community psychologist to be part of social transformation.

### Developing Cultural Humility for Community Partnership

Before traveling to Caquetá, most of the students had hierarchical views of collaboration with the community. Some students may have been uncomfortable with the idea of not having a previous community intervention plan, and others expressed they wanted to contribute to the community as part of their vocation. Despite the good intentions, this perspective holds views of the community as the other in need, to be helped by professional experts. Then, during fieldwork, when the students interacted with people in daily-life activities, they realized former guerrilla members and their families were autonomous. If a partnership was to be constructed, it had to recognize everybody’s potential to equally contribute to the work.

After the first interactions, students started to develop respect and curiosity for the community members’ history, as expressed in the stories compiled in the book and the photo reportage. For example, Daniela, one of the students from group A wrote in her chronicle:

The former guerrilla member’s stories allow me to move from my prejudice towards the unexpected, which manages to appear as a small silver lining. “If I had a gun, you wouldn’t be here”, said one of the ex-combatants, excited about our presence. This phrase summarizes my confusion of a war that was born out of lack of opportunities, of a country that has forgotten about its people and the value of the difference. Behind the AK-47 rifles, responsible for the death of people during decades, human beings wishing for a better life have always existed. (Daniela, 21 years old, 7th semester, individual narrative of the experience)

After the experience, most students had developed a more bidirectional view of community learning and collaboration. For example, during one of the literacy classes they were collaborating with, one of the students managed to overcome the initial frustration of teaching something to an apparently ignorant person:

So, the first time I was there [in the class], I have to admit it humbly, I felt as if I was the one who knew everything, with all the knowledge, I thought I knew all the answers, I have to confess. The next day... I didn’t want to go again [...] but then one of the professors said “you have to go again”, right after I said I didn’t want to go, that I didn’t feel like a good teacher, and then she said I had to go. The next day it was better for me [...] when I let him speak it was beautiful because he showed me a different way of understanding the problem. (Lorena, 9th semester, 20 years old, group A, focus group)

In both groups, this shift was based on the recognition of the other’s expertise and agency, as well as the recognition of one’s limitation of previous knowledge about others. In this process, the experience of being learners of daily-life tasks involved in making a living of a rural community was significant:

Today, we were divided into small committees of about 2 or 3 students, pineapple harvesting, making shoes, removing weed of the coffee crops. The experience was... I had to remove the weeds of the coffee crops and it was a beautiful experience because I was thinking, while I was doing it, that sometimes I take my coffee in the morning and most of the time we don’t know where it comes from and the effort for people to produce the coffee and take it to the city [...]. The day ended with a football match with the community and it was very touching to see how the community has welcomed us and invited us to all their daily activities. (Santiago and Rodrigo, 19 years old, 7th and 8th semester, group B, video diaries)

Most of the group B students experienced a strong dilemma around the former guerrilla members’ values that students did not share. Therefore, more reflective spaces were required during and after fieldwork in Caquetá in order to problematize the possibility of working together while recognizing at the same time different worldviews. In group A, the experience of building partnerships arose easily once the community and the students experienced the “encounter” and the possibility of an alliance:

Talking about our differences, we talked about love stories and even their perceptions about public and private universities. This man, with a child soul, ended up sharing a reflection when I asked him persistently what he thought about us: “You are like 18 different worlds [referring to the students and professors], 18 seeds to harvest, just like us, in order to build a better country to live” [...] What we don’t know is that to have an encounter with the other, we don’t have to erase the differences, but to shorten them. The tension among our differences can work as a hinge to open windows for those who still have them shut. (Sarah, 22 years, 10th semester, group A, individual narratives of the experience)

It is important to highlight that students reported the teachers’ role in developing self-consciousness by modeling and facilitating critical reflection. They valued being recognized as capable of teaching (when they collaborated in the education committee explaining people math and Spanish exercises they had to complete), but also struggled when they found out and had to recognize their own limitations. They also discovered cultural humility in their teachers, who were open to learning from the students and the community in similar terms:

They [a group of community members] confessed that when they heard that people from a private university were visiting, they thought we were going to be “shallow”. This was contradicted when they realized that the two women they had recently talked to in such a natural way, turned out to be the professors. (Kattia, 21 years old, 10th semester, group A, individual narratives of the experience)

In summary, as students interacted with the community people, they started to understand that effective partnership with communities require the recognition of the others’ abilities and knowledge. Instead of a hierarchical approach where professionals are the only experts, students recognized that expertise is a complex concept, and all the participants in the partnership could and should collaborate. This comprehension helped students to develop respect and genuine curiosity toward the community members. This ability to work in partnership with communities from a cultural humility framework is essential to the formation of psychology professionals in the context of peacebuilding.

### Forming Communities of Practice

At the beginning of the course, before traveling to Caquetá, students saw in the experience an opportunity to learn from their peers and professors since they shared similar interests and backgrounds. The students wanted to expand their knowledge about community psychology and about the “reality of the country” through direct experience. As one student stated:

Why did I choose this course? Ever since the professors told us about the idea of the course, it caught my attention because it is an opportunity to “get out of the bubble,” which is Bogota and the University, and to be able to get to know another part of the country’s reality. (Carlos, 19 years old, 5th semester, group A, narratives about expectations)

However, as they traveled and started to interact with the community, they realized that there is no one single reality and that the former guerrilla members could also be part of the community of practice, where they both could learn from one another.

Through dialogue, students became aware of the community knowledge and expertise. Former guerrilla members possessed specific knowledge about farming, shoemaking, and nature, among others, that was useful for their daily-life activities. After some interactions, students realized that learning not only happened in formal educational settings; learning occurred in informal interactions and practices within the community. As students interacted with the community in daily practices, they became aware of the potential of learning *with and from* the community members. For example, one student recalled,

I have always considered myself a person with an important tie to nature. During my childhood, I visited various forest, and considered I had a significant knowledge about nature […] They [former guerrilla members] know how to live in the jungle, and I barely could observe and listen to the forest. However, we both knew how to walk in the jungle (*en el monte*). (Selena, 20 years old, 8th semester, group A, individual narratives of the experience)

As part of the final projects for both courses, the students in collaboration with the community engaged in one project of interest for the community. In December, the students wrote about their experience at the ETCR-HR and some of the stories of former guerrilla members, as a result they published a book titled “Meeting Point. Chronicles of the Encounter.” In June, the students did a photo reportage with former guerrilla members about the intersections between their lives and experiences. In both settings, an editorial committee was created: the professors were the first filter, whose job was to proofread and suggest content adjustments. Teachers encouraged students to work with peers, so students were also editors in a second stage. But the most important one was the third filter: the leaders of the local library as well as the community members that were being portrayed or written about, who read and approved the documents/photos. This exercise was another opportunity to consolidate the collaborative relationships where even the construction of an academic text required self and collective reflection:

Some students had finished their texts, so it was time to show them to the community, so they could be sure that we were telling things as they happened, but also as a way to check if they agreed on what we had written. Our purpose was to leave to the community something transparent and adequate. We went in pairs to show our texts and we were very nervous. It wasn’t easy for us to show community members our texts; we didn’t know if they would like what we wrote. (Renata, 21 years old, 10th semester, group A)

Moreover, most students experienced a paradigmatic shift when they understood there was something important that they could learn from the community members beyond their personal experience. When they co-constructed stories and photo reportages, students gained a comprehension that knowledge goes beyond academic knowledge. To survive in the jungle (*en el monte*), former guerrilla members needed specific knowledge about the weather, natural cycles, animal behavior, and even medicine, that students had not considered before these interactions. As they started to learn from the community, they expanded their community of practice to include community members as significant others, as experts in their own domains. Then, students began to understand how collaborative relationships can consolidate strong and lasting alliances that are able to mobilize social transformation.

### From Peace Agents to Self-Reflective Practitioners

As we have previously mentioned, initially, students’ main goal was to become an example of peacebuilding, to become peace agents as a result of the participation in the experience. Moreover, the narratives of the experience during the 2 weeks presented the complexity of becoming part of a peacebuilding academic project. Moving from a notion of superiority to one of awareness of the value of the community entails an emotional struggle that, in turns, helps them understand multiple realities. Thus, cultural humility appears as the interphase between a state of confusion and the openness to establish dialogical relationships with community members by recognizing the plurality and legitimacy of knowledges. Forming communities of practice is then the final step toward understanding the bidirectional nature of community psychology and the importance of collaboratively developing projects.

It is worth noting that this transition is framed within intentional pedagogical spaces designed to support students throughout their process. The design and execution of the project entailed permanent and fluid communication between the students and the professors, where grades and academic performance were not the main goal; on the contrary, performance was achieved through the levels of self-awareness and the type of connections and bonds they created among themselves and the community:

We were heading towards the restaurant for the last time, in the midst of a heavy rain, and we finished our journey with a self and peer evaluation to fulfill our academic duty. But that was not the most important thing from our last breakfast there [...] the most important thing was our last meeting, where we discussed the strengths and things to improve in the course. We all agreed that the best part of the experience was the overall unremarkable experience we had. (Mauricio and Victoria, group A, daily experience journals)

After the TSL experience, students realized that the essentialized idea of the “other” played an important role in peacebuilding processes in a post-conflict setting. Therefore, some students went back to their context, aware of the responsibility of sharing with other people the multiple stories and practices they had experienced:

So, back home, we [two students from group A] volunteered in a retirement home, in a poetry club for the elderly. We had the book that was written by the former guerrilla members and we told the elderly about it. The grannies are so lovely and cute, and then we started telling them about our experience, through some poems that were part of the book. They were so excited about our experience that the next time, we kept on discussing what had happened to us over there. It was so beautiful. (Miranda, 19 years old, 8th semester, focus group)

This example outlines the ways in which some students have managed to challenge black and white versions of the armed conflict and have started introducing alternative understandings of former guerrilla members. As such, TSL can serve as a platform to trigger conversations about rurality and seemingly distant problems into an urban setting that has been historically indifferent to those stories.

## Discussion

Building peace is a complex process that should involve all members of society, universities and professionals included. Therefore, consolidating a curricular approach toward peacebuilding in universities implies the development of specific competences that enable future psychology professionals to understand the articulation between their discipline and its application in a post-conflict setting. New approaches – such as the historical memory education (Corredor et al., 2018) – highlight the importance of formal and informal settings to promote discussions, debates, and reflections about the structural factors that have promoted violence in the country, as well as to provide space for affective understandings of the contexts and emotions involved in the experience.

The TSL experience we present in this paper is an example of one possible venue to close the gap between rural and urban communities in a divided and polarized country. We argue that intentional pedagogical spaces in real-life scenarios with former guerrilla members can be not only a curricular innovation in terms of training psychologists, but also a peacebuilding strategy for young citizens. Because of the high-intensity dissonance the experience creates in the students (Naudé, 2015), it offers a unique opportunity to generate deep, long lasting personal transformations, which in turn will foster social change (Mezirow, 2000). For this reason, it is important to consider the elements of the intentional pedagogical spaces that provide the foundation for a positive experience in TSL.

First, by intentional pedagogical spaces we refer to the design and implementation of courses on-site where there is space for an encounter or a high-intensity dissonance. In a post-conflict setting such as Colombia, the focus has been on teaching history of the armed conflict and creating debates in class about the country current situation (Gómez-Suárez, 2017; Corredor et al., 2018; Díaz-Gómez et al., 2019; Oettler and Rettberg, 2019). However, these pedagogical strategies are framed during class or using specific activities with special guests on campus. In the current study, one of the critical aspects that triggered a contextual understanding of the socio-economic situation of people in a reincorporation process was the possibility to spend time with them in informal, daily-life scenarios in context. This is in line with studies on the importance of service-learning experiences in the psychology curriculum, especially on the benefits of community partnerships to create opportunities for critical reflection (Bringle et al., 2016).

Second, cultural humility offers a framework to educate psychologists with intercultural competences to work in post-conflict scenarios. Some characteristics of cultural humility include: cultural consciousness, cultural limitations, self-reflection on one’s privilege and power (Fischer-Borne et al., 2014), awareness about the impossibility to fully understand other worldviews (Hook and Davis, 2019), the recognition of the others’ knowledge and expertise (Isaacson, 2014), and respect and curiosity toward their culture and history (Hook and Davis, 2019). We recognize that one 2-week experience is not enough to fully develop cultural humility, but as students experienced paradigmatic shifts and high-intensity dissonances, they showed development of various elements of cultural humility in progress.

Findings suggests that self-reflection, cultural consciousness, and recognition of others knowledge played a role on the quality of the relationships between teachers, students, and the community. This finding agrees with research on cultural humility and how it facilitates partnership in psychology scenarios as it works for therapeutic alliances in clinical settings (Abbot et al., 2019). Even more, the centrality of emotional struggles and dilemmas related to preconceptions of the other indicates that a multicultural orientation is key for peacebuilding interventions whose goal is to train self-aware and critical professionals.

Finally, critical reflection plays an essential role in the transformative process before, during, and after the field experience. This curricular design involves a critical approach to education, where the relationship between students and professors is horizontal, and safe spaces are guaranteed in order to deal with the emotional struggle students face during the TSL experience. This supporting relationship is consistent with research about critical reflection and metacognition as a positive way to foster reflection, be consciously aware of biases and prejudices, and therefore promote cultural humility (Sánchez et al., 2019). In this study, safe spaces for discussion were provided both orally and in written through daily meetings with the students, and with the daily recordings or texts about their experience. The creation of the editorial committees with the community was an additional, non-planned space that further created room for self-awareness and reflection about the writing process.

In conclusion, building peace is a complex process that requires the commitment of all sectors of society. If universities are to contribute to this endeavor, complex and innovative solutions should be proposed with the input and collaboration of those who have been involved in the process. TSL offers a pedagogical strategy to develop intercultural competences in post-conflict settings in future psychology professionals. The social impact of these interventions goes beyond the profession as it serves to close the gap between rural and urban contexts, and fosters dialogues between former guerilla members and civilians that were unthinkable in the past.

### Strengths and Limitations of the Present Study

This study is drawn from two on-site courses held during two intensive weeks. Analysis of students’ transformation and self-reflection can only be extended to the 4 months after the experience, where focus groups were held. Additionally, student characteristics, such as participating previously in a Student Research Group, could have an influence on the willingness and openness to participate in this type of experiences.

Nevertheless, it is worth noting that this study presents a rigorous systematization of the self-reflection processes of undergraduate psychology students, as well as documentation of pedagogical strategies used to develop cultural humility in students. By combining not only written texts but oral experiences, the study delves deep into different forms of expression of self-awareness and the development of critical consciousness.

### Future Research

The development of cultural humility is a lifelong process (Sánchez et al., 2019) that can start during an intensive 2 weeks course but needs time to settle in. The results of this study suggest that openness to learn from others and a self-reflection process indeed took place during and after the course. Future studies need to examine whether the intercultural competences gained in this experience are transferred to professional practice.

## Data Availability Statement

The datasets generated for this study are available upon request to the corresponding author.

## Ethics Statement

The present study is part of a larger research project approved by the Ethical Committee of Universidad de La Sabana in session Number 77 on August 14, 2019. All participants provided oral informed consent, which was audio recorded. No explicit informed consent was collected from the parents/legal guardians of non-adult participants, as all participants were above the age of 18. Written consent can be provided if required.

## Author Contributions

LF prepared the data sets used for data analyses, wrote the justification of the study focused on Colombian’s current socio-historical situation, “Methods”, “Results”, and “Discussion”. LT-C performed major revisions of the document; described the theoretical framework with an emphasis on Transformative Service-Learning and cultural humility; connected the sections of the manuscript; and wrote the methods, results and discussion. NR was in charge of the literature review on cultural humility, wrote the methods, the results and the discussion. LF and NR were in charge of data collection in the two courses. All the authors participated in the team-based analysis and construction of the sections “Results” and “Discussion.”

### Conflict of Interest

The authors declare that the research was conducted in the absence of any commercial or financial relationships that could be construed as a potential conflict of interest.
